# Job Insecurity and Mental Health: The Moderating Role of Coping Strategies From a Gender Perspective

**DOI:** 10.3389/fpsyg.2019.00286

**Published:** 2019-02-18

**Authors:** Sara Menéndez-Espina, Jose Antonio Llosa, Esteban Agulló-Tomás, Julio Rodríguez-Suárez, Rosana Sáiz-Villar, Héctor Félix Lahseras-Díez

**Affiliations:** ^1^Department of Psychology, University of Oviedo, Oviedo, Spain; ^2^Department of Health Sciences, International University of La Rioja (UNIR), La Rioja, Spain

**Keywords:** job insecurity, mental health, coping strategies, moderating role, gender perspective, gender

## Abstract

Job insecurity is a growing phenomenon, typical of an employment context characterised by high rates of temporary work and unemployment. Previous research has shown a direct relationship between job insecurity and mental health impairment. The present analysis goes into this relationship in depth, studying the moderating role of coping strategies and predicting that men and women implement different types of strategies. A sample of 1.008 workers is analysed, 588 women and 420 men. The Tobin CSI scale was used to analyse the coping strategies, in addition to JIS-8 to assess job insecurity, the MOS Perceived Social Support Survey and the GHQ-28 test to evaluate mental health. Then, a hierarchical linear regression was designed to study the moderating role of 8 coping strategies of job insecurity and 4 mental health subscales in men and women, separately. Results illustrate that coping strategies play a moderating role in the relationship between job insecurity and mental health. However, the aggravating role of disengagement coping strategies is more relevant than the buffering role of engagement strategies. On the other hand, women implement a greater number of coping strategies, with more positive results for mental health. Also, in the relationship between job insecurity and mental health the most important strategies are the ones related to social interaction inside and outside an organisation, and these are the main ones used by women. It therefore follows that strengthening rich social relationships inside and outside the working environment is a guarantee of well-being.

## Introduction

The concept of job insecurity was first defined in the eighties as “the perceived powerlessness to maintain desired continuity in a threatened job situation” (Greenhalgh and Rosenblatt, 1984, 438). Since then, there is a trend to abandon the Keynesian job model, characterised by stable jobs and professional careers, in favour of a more flexible labour market that generates more unstable and precarious working conditions (Burchell et al., 2014; Kim and von dem Knesebeck, 2015; Nielsen et al., 2015). Consequently, the interest in studying job insecurity has gradually increased, especially in the aftermath of the 2008 economic crisis, as since then, the number of research studies related to this topic has multiplied exponentially (Llosa et al., 2018). Moreover, in countries and/or periods in which unemployment figures rise, a greater fear of job loss is observed among workers (Keim et al., 2014). In this sense, job insecurity is a concept very much linked to precarious work, which highlights the worker’s perception of his/her employment situation. Thus, the individual’s subjectivity is linked to socioeconomic circumstances resulting in a psychosocial phenomenon of precarious work. Job insecurity has been studied as a stressor in the working environment (Greenhalgh and Rosenblatt, 1984). As a generator of stress, there is evidence of manifold negative consequences on workers’ well-being and mental health (Cheng and Chan, 2008; De Witte et al., 2016; Lee et al., 2018; Llosa et al., 2018), related both to depressive disorders (Blom et al., 2015; Kim et al., 2017), and to anxiety (Boya et al., 2008). It also has an impact on physical health, such as general physical well-being (Henseke, 2018) or heart diseases (Schnall et al., 2016).

This analysis aims to accurately identify the role played by coping strategies between men and women, when minimising or buffering the negative impact of precarious labour relations on mental health. In this manner, studying coping strategies represent one of the most accessible intervention pathways in any environment.

To approach coping strategies, this study is grounded in the theories of Lazarus and Folkman. These authors define coping strategies as “the constantly changing cognitive and behavioural efforts implemented to manage specific internal and external demands that are appraised as exceeding the resources of the person” (Lazarus and Folkman, 1984, 141). Edwards (1988) integrated this definition into the cybernetic theory of stress, considering coping as an effort to reduce or eliminate the negative effects of stress in a person’s well-being. According to several authors, it is what the observer sees, rather than the characteristics of the situation, that determines whether or not the circumstances are evaluated as stressful (Lazarus and Folkman, 1984; Roskies et al., 1993; Lazarus, 1996).

The most widespread classification of coping strategies identifies two different types of strategies that may be implemented when faced with a stressful circumstance: problem-focused strategies and emotion-focused strategies (Lazarus and Folkman, 1984). The former, geared toward changing the source of stress, generally come about when the source is perceived as subject to change. Emotion-focused strategies, in contrast, consist of regulating the emotional response to the problem, and they are more likely to be implemented when the situation is evaluated as unchanging. In a similar line, Pinquart and Silbereisen (2008) observed that depressive symptoms resulting from different stressful circumstances decreased when implementing one or other type of strategies, depending on whether the situation was perceived as unchanging or not. A third class has also been included, namely, the avoidance strategies, consistent in distancing themselves from the problem, both physically and cognitively (Skinner et al., 2003).

In addition to this general classification, other strategies have been identified when faced with a stressful circumstance. Tobin et al. (1989) reviewed the primary strategies defined by previous authors to build the Coping Strategies Inventory (CSI), proposing eight strategies: *Self-Criticism* (cognitive strategies focused on criticising oneself and blaming oneself), *Problem Solving* (cognitive and behavioural strategies designed to eliminate the source of stress by changing the situation), *Cognitive Restructuring* (altering the meaning of the source of stress to perceive it as less threatening, for example, seeing the positive side), *Express Emotions* (exteriorising emotions and feeling), *Social Support* (seeking emotional support from family and friends), *Wishful Thinking* (cognitive strategies that reflect the desire for reality to be better), *Social Withdrawal* (isolating oneself socially from other people), and *Problem Avoidance* (denying the problem and avoiding thoughts or behaviours related to the source of stress). According to these authors, Self-Criticism, Wishful Thinking, Social Withdrawal and Problem Avoidance are strategies defined as disengagement strategies, and the others would be engagement strategies. In the scale adapted to Spanish, Cano-García et al. (2007) confirmed that the same strategies were present in other measurement tools such asWOC or COPE.

### Coping Strategies and Job Insecurity

Several studies have demonstrated the moderating role of coping strategies in the relationship between job insecurity and mental health, although analysing the type of strategies under a general classification (emotion-focused or problem-focussed). Bosmans et al. (2015) conclude, after interviewing a group of workers from employment agencies, that the strategies implemented worsen or buffer mental health impairment due to precarious work. According to Richter et al. (2013) and Probst and Jiang (2016), emotion-focused strategies are the most effective ones to reduce the negative consequences of job insecurity on workers’ well-being. However, problem-focused strategies would not always be effective because it is an uncontrollable stressor (Vander Elst et al., 2014). Avoidance strategies, in turn, aggravate the problem. On their part, Cheng et al. (2014) establish that a set of strategies, which they called active strategies (symptom reduction, accommodation, changing the situation and devaluation), buffered the effects of job insecurity in some variables of psychological well-being in both work and home domains. Furthermore, the authors underscore that the avoidance strategy, catalogued as inactive, induces the opposite effect. These authors highlight the need to use a more specific strategic approach, as they do in their study, instead of just focusing on traditional global categories: emotion-focused or problem-focused. Thus, our research aims to make this approach where the specific strategies that influence the relationship between job insecurity and mental health are considered.

### Job Insecurity and Gender

The social dimension of job insecurity means that it is not a phenomenon alien to gender inequalities. Although recent research studies establish that men and women have similar levels of fear of job loss (Rigotti et al., 2015), it has not been analysed whether the use of these strategies affects health in a different way in men and women when the stressor is the anticipation of job loss. Previous studies have focused on gender differences in terms of strategies implemented when faced by stressful events in general. For example, it has been found that women tend to use more strategies than men, both problem-focused and emotion-focused strategies, and they seek more emotional support (Tamres et al., 2002). At the same time, Matud (2004) underscores that the social support strategy and the problem avoidance strategy are specific strategies linked, to a great extent, to women. However, strategies should play an efficient role, namely, to reduce stress, and in this sense, Gattino et al. (2015) found that emotional and instrumental social support strategies are the ones that have the greatest influence on women’s quality of life. However, in the case of men, this well-being is significantly impaired if they use self-criticism as a strategy. Such differences would respond to socialisation on the basis of some pre-established roles, which have two effects: on the one hand, they mean that people are taught different coping styles depending on the allocated gender (Nelson and Burke, 2002); and, on the other hand, generally speaking, men and women do not cope with the same kind of stressors, so the strategies implemented are also different (Banyard and Graham-Bermann, 1993; Matud, 2004). However, our study analyses the same kind of stressor, in such a manner that we control this second effect. Knowing this relationship between coping strategies and gender, in our study we want to analyse how their efficacy varies in men and women.

### Social Support as a Differential Coping Strategy Between Men and Women

In the study of job insecurity, social support has been one of the main coping strategies addressed. Social support develops differently in men and women. It is defined as “a social network’s provision of psychological and material resources intended to benefit an individual’s ability to cope with stress” (Cohen, 2004, 676). Several studies have demonstrated that social support decreases the negative effects of job insecurity (Lim, 1996; Snow et al., 2003; Näswall et al., 2005a; Sora et al., 2011), and this strategy is used more often, and also more efficiently, by women (Matud, 2004). In this manner, in our study social support could be more effective in women when they face job insecurity.

There are different types and sources of social support, although in our study two measures of this variable are used: on the one hand, social support as a coping strategy, and, on the other hand, perceived social support (PSS), which refers to the emotional, instrumental and affective resources that a person has (Sherbourne and Stewart, 1991). Thus, although PSS is related to the way in which people feel supported and protected socially, the social support strategy refers to the use we make of those networks. In this sense, Cohen and Wills (1985) suggested that PSS has direct effects on well-being, whereas received social support has protective effects against stress. According to Lakey and Heller (1988), the latter has an influence on coping behaviours, whereas the former has an impact on processes of cognitive evaluation. However, the support specificity model proposed by Cohen and McKay (1984) establishes that for social support to be effective, it should be accommodated to the specific stressor and circumstance. This different effect could occur in coping with job insecurity, so it will also be testedin this study.

### This Study

The research question posed is which coping strategies reduce the impact of job insecurity on mental health, and whether there are gender differences between them. Therefore, the main objective is to perform a comparative study between men and women to analyse the moderating role of coping strategies in the relationship between job insecurity and mental health in a sample of Spanish workers. To this end, the following hypotheses are formulated:

H1: Job insecurity is related to higher scores on health scales (somatic symptoms, anxiety, social dysfunction and depression) in men and women alike.
H2: Engagement coping strategies are related to lower scores on health scales, whereas disengagement ones are related to higher scores, in women and men.
H3: Coping strategies play a moderating role in the relationship between job insecurity and health in a different way in men and women.
H4: Coping strategies related to social interaction play a more relevant role, in terms of moderation between job insecurity and mental health, in women than in men.
H5: Perceived social support plays a moderating role in the relationship between job insecurity and mental health.

To date, there are no research studies that provide a specific and detailed approach regarding coping strategies related to the relationship between job insecurity and mental health. This study aims, therefore, to compile different designs used in previous studies to provide new and more practical knowledge of interventions when job insecurity occurs. Following the recommendations of Richter et al. (2013) and Cheng et al. (2014), the aim of this study is to provide knowledge about the moderating role played by specific coping strategies in terms of the relationship between job insecurity and health. To guide the implications of our results toward a practical intervention approach, the major strategy taxonomies are not analysed (problem-focused and emotion-focused, or engagement and disengagement strategies). The analysis of PSS has also been included to verify the relationship between having this kind of support and using it effectively as a strategy when faced with a stressor such as job insecurity. Finally, gender differences are examined to establish whether social processes leading to gender inequality have an influence on the strategies implemented to cope with job insecurity, and, if so, explain such deviation.

## Materials and Methods

### Participants

The sample includes 1008 participants belonging to the Spanish population. Of the total, 420 are men (41.7%) and 588 women (58.3%), aged between 18 and 63 years of age, with a mean of 36.05. At the time of the study, all the subjects were working. To know the profile of the workers participating in the study, the working characteristics and conditions of the sample are compiled in Table 1.

**Table 1:** Percentage distributions, means, and Standard Deviations of labour profiles of the sample.

	Total	Men	Women
**Occupation**			
Managers	4.8	6.9	3.2
Professionals	13.6	14.5	12.9
Technicians and associate professionals	19.5	18.58	20.1
Clerical support workers	6.9	5.2	8.2
Service and sales workers	34.1	27.6	38.8
Skilled agricultural, forestry and fishery workers, craft and related trades workers, machine operators	8.9	17.8	2.4
Armed forces occupations	1.4	3.1	0.2
Elementary occupations	10.8	6	14.3
**Type of contract**			
Indefinite	43.6	50.7	38.4
Temporary	45.7	39.7	49.9
No contract	10.8	9.5	11.7
**Type of day**			
Full day	73.2	77.9	69.9
Part-time day	26.8	22.1	30.1
**Tenure (months)**	91.6(121.4)	110.4(136.4)	76.9(106.1)
**Age**	36(12.2)	36.6(13.2)	35.5(11.5)


### Procedure

The procedure applied was causal and quota sampling. To this end, the profile of participants required for the study was defined and, then, the people voluntarily completed a self-administered questionnaire. Participants were informed that the data would only be used for research purposes. This research study follows the protocols and requisites of the Ethics Committee of the Department of Psychology of the University of Oviedo (Spain).

### Materials

#### Coping Strategies

The CSI by Tobin et al. (1989) was used to measure the coping strategies. The Spanish version has been validated by Cano-García et al. (2007), and includes 40 items in a 5-point Likert scale format. This scale permits measuring 8 specific coping strategies: Problem Solving (α = 0.82), Self-Criticism (α = 0.94), Express Emotions (α = 0.89), Wishful Thinking (α = 0.78), Social Support strategy (α = 0.89), Cognitive Restructuring (α = 0.83), Problem Avoidance (α = 0.72), and Social Withdrawal (α = 0.81). The authors who validated the Spanish version highlight that these strategies are similar to the ones used in other tools, such as the ways of coping (WOC) (Folkman and Lazarus, 1980) and the COPE (Carver et al., 1989), and that they may be accepted as generic strategies to cope with stressful circumstances.

#### Mental Health

The General Health Questionnaire GHQ-28 (Goldberg and Hillier, 1979) was used, adapted to the Spanish population by Retolaza Balsategui et al. (1993). This version includes 28 items and it has a Likert type 4 alternative response format. It measures the general mental health condition in the non-clinical population, obtaining both a general score and four specific scores based on subscales: somatic symptoms, anxiety and insomnia, social dysfunction and severe depression. This scale is widely used in research in psychology with a 0.90 reliability rate in the Spanish adaptation. Subscales are used as independent variables to understand the subject matter in greater detail.

#### Job Insecurity

JIS-8 or 8-item version Job Insecurity Scale, developed by Pienaar et al. (2013) was used. It has a Likert type 5 response alternative format. It offers a global score and it also measures two dimensions of job insecurity: the cognitive dimension, made up of the first 4 items, and the affective dimension, which includes the 4 remaining items. It has been validated to the Spanish population by Llosa et al. (2017), resulting in a reliability score of 0.88 for the global score, which has been used in this study.

#### Perceived Social Support

This has been measured using the MOS Scale of PSS (Sherbourne and Stewart, 1991), validated to the Spanish population by Revilla Ahumada et al. (2005) with an internal consistency close to 1. It includes 20 items: one with open-ended response and 19 Likert type from 1 to 5.

### Data Analysis

A hierarchical linear regression was performed to study the moderating role of the eight coping strategies between job insecurity and the four mental health subscales. Following recommendations by Cohen et al. (2003), the predictive variable (job insecurity) and the moderators (each of the coping strategies and PSS) were centred. Thus, the interpretation of the results is facilitated and multicollinearity among the predictive variables is reduced (Aiken et al., 1991). The regression analysis was performed in four stages: first, the job insecurity variable was introduced, based on the global score on the JIS-8 scale. Secondly, the second variable of PSS was added. Then, the eight coping strategies were introduced, and finally, the interaction between job insecurity and each of the coping strategies, as well as the variable of PSS.

## Results

Table 2 shows the means, standard deviations and correlations of all the variables included in the study, segregated by genders. The regression results are shown in Table 3 for men and Table 4 for women.

**Table 2:** Correlations, means, and Standard Deviations of the variable included in the study by gender.

	1.	2.	3.	4.	5.	6.	7.	8.	9.	10.	11.	12.	13.	14.
(1) Job insecurity	–	–0.20**	–0.19**	0.01	–0.10*	0.05	–0.17**	–0.27**	–0.11**	0.04	0.16**	0.22**	0.17**	0.21**
(2) Perceived social support	–0.19**	–	0.34**	–0.10**	0.25**	–0.00	0.58**	0.34**	0.07	–0.27**	–0.24**	–0.25**	–0.20**	–0.30**
(3) Problem solving	–0.15**	0.32**	–	–0.08*	0.44**	0.13**	0.48**	0.52**	0.02	–0.15**	–0.16**	–0.15**	–0.28**	–0.22**
(4) Self-criticism	0.17**	–0.13**	–0.15**	–	0.08*	0.34**	–0.05	–0.02	0.08*	0.43**	0.23**	0.27**	0.19**	0.29**
(5) Express emotions	0.05	0.14**	0.20**	0.12*	–	0.30**	0.56**	0.38**	0.05	–0.19**	–0.03	0.01	–0.11**	–0.12**
(6) Wishful thinking	0.14**	0.10*	0.07	0.31**	0.31**	–	0.15**	–0.01	–0.01	0.24**	0.11**	0.21**	0.14**	0.18**
(7) Social support	–0.13**	0.39**	0.31**	–0.01	0.46**	0.25**	–	0.48**	0.06	–0.35**	–0.22**	–0.18**	–0.21**	–0.23**
(8) Cognitive restructuring	–0.09	0.24**	0.45**	0.06	0.34**	0.18**	0.42**	–	0.37**	–0.10*	–0.23**	–0.23**	–0.32**	–0.25**
(9) Problem avoidance	0.04	0.05	–0.03	0.17**	0.14**	0.17**	0.06	0.39**	–	0.28**	–0.05	–0.11**	–0.12**	–0.02
(10) Social withdrawal	0.22**	–0.18**	–0.15**	0.52**	–0.10*	0.28**	–0.24**	–0.03	0.36**	–	0.18**	0.21**	0.19**	0.35**
(11) Somatic symptoms	0.24**	–0.20**	–0.20**	0.23**	–0.00	0.16**	–0.11*	–0.16**	–0.01	0.23**	–	0.65**	0.49**	0.44**
(12) Anxiety and insomnia	0.24**	–0.24**	–0.18**	0.26**	–0.05	0.17**	–0.20**	–0.21**	–0.02	0.35**	0.68**	–	0.51**	0.54**
(13) Social dysfunction	0.21**	–0.18**	–0.27**	0.17**	–0.10*	0.13**	–0.15**	–0.16**	0.02	0.27**	0.44**	0.51**	–	0.55**
(14) Severe depression	0.28**	–0.28**	–0.23**	0.31**	–0.04	0.16**	–0.15**	–0.18**	0.04	0.40**	0.46**	0.63**	0.51**	–
Men mean	19.44	79.10	17.74	11.68	12.32	15.58	15.54	15.06	11.32	10.89	4.53	5.31	6.90	1.88
Men SD	7.17	14.50	4.39	4.72	4.38	5.20	5.24	4.07	3.92	4.39	3.69	4.47	2.70	3.34
Women mean	21.88	78.65	18.25	11.62	14.67	17.42	17.28	15.07	10.92	10.48	6.54	6.96	7.63	2.31
Women SD	6.94	15.32	4.26	4.79	4.81	4.97	5.28	4.44	4.01	4.36	4.08	4.92	2.91	3.50


*Women (*N* = 576) above the diagonal; men (*N* = 415) below de la diagonal; ^∗^*p* < 0.05; ^∗∗^*p* < 0.01.*

**Table 3:** Results of hierarchical regression analysis for job insecurity, coping strategies and GHQ-28 subscales for men (standardised coefficients).

	Variables	Somatic symptoms	Anxiety and insomnia	Social dysfunction	Severe depression
Step 1	Job insecurity (JI)	0.241**	0.242**	0.210**	0.280**
	Δ*R*^2^ (*R*^2^ Adj)	0.058**(0.056**)	0.059**(0.056**)	0.044**(0.042**)	0.079**(0.076**)
Step 2	Perceived social suppor (PSS)	–0.164**	–0.205**	–0.144**	–0.243**
	Δ*R*^2^ (*R*^2^ Adj)	0.026**(0.080**)	0.041**(0.095**)	0.020**(0.059**)	0.057**(0.131**)
Step 3	Problem solving (PS)	–0.090	–0.017	–0.191**	–0.074
	Self-criticism (SC)	0.104	0.074	0.004	0.096
	Express Emotions (EE)	0.020	0.028	–0.068	0.003
	Wishful thinking (WT)	0.121*	0.122*	0.116*	0.067
	Social support (SS)	0.008	–0.051	0.012	0.068
	Cognitive restructuring (CR)	–0.103	–0.133*	–0.030	–0.109
	Problem avoidance (PA)	–0.057	–0.104	–0.054	–0.057
	Social withdrawal (SW)	0.099	0.257**	0.188*	0.295**
	Δ*R*^2^ (*R*^2^ Adj)	0.075**(0.138**)	0.133**(0.213**)	0.098**(0.142**)	0.141**(0.258**)
Step 4	JIxPS	0.006	–0.028	0.037	–0.027
	JIxSC	0.091	–0.090	–0.035	–0.061
	JIxEE	–0.020	–0.043	–0.058	0.005
	JIxWT	–0.070	–0.012	0.076	–0.015
	JIxSS	0.074	–0.011	0.096	0.082
	JIxCR	–0.078	–0.114	–0.105	–0.030
	JIxPA	0.078	0.066	0.070	–0.065
	JIxSW	0.045	0.106	0.126	0.273**
	JIxPSS	–0.048	0.068	–0.054	0.012
	Δ*R*^2^ (*R*^2^ Adj)	0.030(0.150**)	0.024(0.221**)	0.034(0.158)	0.043**(0.286**)


*N = 585; ^∗^*p* < 0.05; ^∗∗^*p* < 0.01; Δ*R*^*2*^ = Change in percentage of variance explained; *R*^*2*^ Adj = Adjusted percentage of variance explained.*

**Table 4:** Results of hierarchical regression analysis for job insecurity.

	Variables	Somatic symptoms	Anxiety and insomnia	Social dysfunction	Severe depression
Step 1	Job insecurity (JI)	0.162**	0.223**	0.174**	0.213**
	Δ*R*^2^ (*R*^2^ Adj)	0.026**(0.024**)	0.050**(0.048**)	0.030**(0.029**)	0.45**(0.044**)
Step 2	Perceived social suppor (PSS)	–0.218**	–0.223**	–0.177**	–0.274**
	Δ*R*^2^ (*R*^2^ Adj)	0.045**(0.068**)	0.048**(0.094**)	0.030**(0.057**)	0.072**(0.114**)
Step 3	Problem solving (PS)	–0.010	–0.032	–0.161**	–0.059
	Self-criticism (SC)	0.180**	0.174**	0.107*	0.151**
	Express Emotions (EE)	0.106*	0.115*	0.022	–0.015
	Wishful thinking (WT)	0.019	0.100*	0.087	0.071
	Social support (SS)	–0.106	–0.048	0.005	0.051
	Cognitive restructuring (CR)	–0.150*	–0.102	–0.172**	–0.107*
	Problem avoidance (PA)	–0.004	–0.087*	–0.080	–**0**.039
	Social withdrawal (SW)	0.036	0.085	0.098	0.230**
	Δ*R*^2^ (*R*^2^ Adj)	0.075**(0.132**)	0.105**(0.188**)	0.119**(0.165**)	0.131**(0.235**)
Step 4	JIxPS	0.082	–0.007	0.040	0.007
	JIxSC	0.017	–0.049	–0.010	0.005
	JIxEE	–0.086	–0.053	–0.023	0.002
	JIxWT	0.000	–0.017	–0.117*	0.028
	JIxSS	0.044	0.135*	0.130*	0.117
	JIxCR	0.012	0.066	–0.036	–0.040
	JIxPA	–0.097*	–0.077	–0.014	–0.021
	JIxSW	0.073	0.089	0.125*	0.136**
	JIxPSS	0.028	–0.065	–0.058	–0.020
	Δ*R*^2^ (*R*^2^ Adj)	0.019(0.138**)	0.014(0.190**)	0.020(0.172**)	0.019(0.243**)


*Coping strategies and GHQ-28 subscales for women (standardised coefficients). N = 585; ^∗^p < 0.05; ^∗∗^p < 0.01; ΔR^2^ = Change in percentage of variance explained; R^2^ Adj = Adjusted percentage of variance explained.*

It is observed that job insecurity (JI) has direct effects on the different areas of mental health evaluated, in men as well as in women. This variable explains between 4 and 8% of the variance in somatic symptoms, anxiety and social dysfunction in men, and in this group it shows a greater weight in depression (β = 0.280). In women, it explains between 2 and 5% in the same variables, and its weight is greater in anxiety and insomnia (β = 0.223). PSS is also statistically significant in all the dependent variables, as well as in both samples, increasing the explained variance percentage for all of them.

In terms of the group of strategies, analysed in stage 3, the main difference between the groups is the existence of a greater variety of statistically significant strategies in the subscales of the female sample. Thus, self-criticism (SC) is the most relevant variable in women, as it is significant in more mental health scores. It is followed by cognitive restructuring (CR) and express emotions (EE). In men, the strategies that are significant in more mental health subscales are wishful thinking (WT) and social withdrawal (SW). If we consider the dependent variables with a greater number of predictive strategies, in the case of men, they are anxiety and insomnia, and social dysfunction, whereas in women the four subscales show a similar influence, anxiety and insomnia being altered by a greater number of strategies.

As for stage 4, where the moderating role of strategies between job insecurity and health subscales is studied, this relationship is only observed in some strategies. There are certain differences between the groups. In men, only problem solving (PS) seems to interact when it is related to job insecurity and severe depression. In women, all the dependent variables are affected: problem avoidance (PA) increases somatic symptoms when it interacts with job insecurity (β = -0.097); anxiety and insomnia decrease with the social support strategy (SS) (β = 0.135); social dysfunction is reduced with the social support strategy (SS) (β = 0.130) and problem solving (PS) (β = 0.125), and it gets worse with wishful thinking (WT) (β = -0.117). Finally, problem solving (PS) is moderated with severe depression (β = 0.136), as occurs in men. No interaction has been observed between the PSS variable (PSS) and job insecurity that is statistically significant in the mental health variables.

## Discussion

The aim of this study was to identify coping strategies capable of buffering the impact of job insecurity on different mental health areas and determine whether or not there were gender differences. The results provide relevant knowledge to scientific literature related to the study of job insecurity, as well as the practical implications to reduce the effects of this phenomenon on workers’ mental health.

In general, it has been found that women possess a greater variety of coping strategies that play a moderating role between job insecurity and mental health, whereas men only have one, social withdrawal, which is also significant in the female group. The most relevant strategies to cope with job insecurity and to prevent the development of mental disorders are social withdrawal and social support. Moreover, it is observed that the role of disengagement coping strategies is more relevant than the role of engagement coping strategies in terms of a moderating effect. The most relevant findings, however, are linked to strategies focused on social interaction, a type of strategy used mainly by women. This shows that strategies related to the collective scale are more useful than those developed individually, which should guide organisational interventions.

Results confirm that job insecurity is a relevant stressor, reinforcing the conclusions obtained in previous studies that also showed these negative consequences of job insecurity on mental and physical health (De Witte et al., 2016; Schnall et al., 2016; Kim et al., 2017; Llosa et al., 2018). This study illustrates that it is a phenomenon that has an impact on workers’ mental health in its different indicators, in men and in women. Thus, there is full compliance with H1.

Regarding coping strategies, its direct relationship with health is also confirmed. Moreover, there is a greater variety in terms of the number and types of strategies that have an effect on women’s health, thus fully complying with H2. This result is similar to the one found by other research studies such as the study by Tamres et al. (2002), although these authors referred to general stressors, while we focus on a specific source of stress, namely, job insecurity. Express emotions appears to be a relevant strategy, as these authors had shown, as well as Matud (2004), because express emotions and social support are strategies that are implemented mostly by women to cope with stressful circumstances. In this group, it has been observed that self-criticism is the emotional reaction that impairs mental health the most, contrasting with the findings of Gattino et al. (2015), who concluded that this strategy had a greater impact on men’s quality of life. In our case, social withdrawal is the strategy with the most significant effect on men’s mental health.

H3 and H4 refer to the moderating role of coping strategies in the relationship between job insecurity and mental health. H3 is complied with, as a different effect has been found between men and women, that is, neither the number nor the type of significant strategies are the same in both genders. Just as there is a greater number of coping strategies directly related to women’s health, it was observed that this relationship is maintained in their moderating role. In the case of men, the only strategy that worsens severe depression symptoms when related to job insecurity is the social withdrawal strategy. In women, avoidance and wishful thinking play a moderating role, but social support and social withdrawal strategies are more relevant because they are related to at least two mental health areas. Therefore, H4 is partially complied with. These latter strategies –social support and social withdrawal-were previously associated with female coping (Matud, 2004). In terms of PSS, this variable does directly have a relevant and positive effect on mental health. However, this variable does not act as a buffer for the consequences of job insecurity, and therefore, H5 is not complied with.

Although the aim of the study was to analyse specific strategies, an interesting result has been found with regard to its general classification. The same pattern is observed in terms of the general effect of the strategies on health and in their moderating role with respect to job insecurity: the aggravating effect of disengagement strategies is greater than the buffering effect of the engagement strategies. The only beneficial strategy for mental health to cope with job insecurity is the social support strategy. In contrast, when comparing this strategy with the results related to PSS, a discrepancy is found. Noteworthy is the difference between the PSS (the perception of having a support network) and the social support strategy (not only the existence of a support network, but that the person uses it).

Perceived social support has a direct impact on the mental health of both men and women, whereas the social support strategy does not. However, to mitigate the effects of job insecurity, the relationship is inverted: social support as a strategy acts as a buffer in women, whereas PSS does not fulfil that role in any of the groups. These results confirm the hypothesis of Cohen and Wills (1985), who explained a functioning and role for each type of support that was similar to the one we have obtained. Thus, to cope with job insecurity, it does not suffice to have a social support network, but rather, individuals should actively resort to it to cope with the analysed stressor. This could be explained by the family being an important source of social support, although having family responsibilities is a risk factor for the development of job insecurity (Sverke and Hellgren, 2002).

With regard to the discrepancy between men and women, a possible explanation could result from the relationship between gender roles and strategies. There is a path of study on coping strategies that states that these are more effective when a gender implements strategies typical of the roles in which they have socialised (Lengua and Stormshak, 2000). According to this theory, women would be more successful when using more emotional strategies, including social support, and men when using strategies more focused on action (Nelson and Burke, 2002; Gattino et al., 2015). However, we have seen that, to cope with job insecurity, in the case of men there are no more efficient strategies, the withdrawal strategy being the only relevant one, which implies rejecting social support. We have observed that this is related to the development of severe depression, especially in men. On the other hand, one of the characteristics of job insecurity is that it is a non-controllable stressor (Vander Elst et al., 2014),and therefore, the strategies that are more focused on problem solving, more widely used by men, would not be more beneficial for well-being (Richter et al., 2013; Probst and Jiang, 2016).

Thus, the efficacy of the social support strategy in women reinforces the idea that socialisation, depending on differentiated gender roles, has an effect on the types of strategies implemented when faced with different circumstances. But, education in gender roles, which trains us in different WOC with situations, limits the adaptation of the most efficient strategies to each event. For example, if men receive an education that is less focused on emotional strategies, it will be more difficult for them to find the necessary resources to achieve successful coping when faced with stressors such as job insecurity.

In short, we have found that job insecurity is a phenomenon that requires more than mere individual coping strategies to buffer its negative impact on mental health. These results perfectly illustrate the important social dimension of job insecurity, similarly to Lim (1996) and Näswall et al. (2005b) who found that the social support obtained both at the workplace and outside the workplace had a positive impact on different areas of workers’ well-being when they developed job insecurity. Authors such as Patterson (2003) and Perreault et al. (2017) obtained similar results regarding the relationship between social support and stress in general. This approach could be associated with the study by Dunahoo et al. (1998), which suggests paying greater attention to the prosocial-antisocial axis around which the reactions in the event of a stressful circumstance can be categorised.

There is a wide array of research studies related to the multilevel or psychosocial dimensions of job insecurity. This phenomenon is influenced by socioeconomic factors, both in terms of their occurrence and their consequences (Keim et al., 2014; Jiang and Probst, 2017; Shoss, 2017; Lee et al., 2018). Even the implementation of strategies such as the intention to change jobs, studied in the context of job insecurity (Stiglbauer et al., 2012), depends on the employability perceived by the individual (Balz and Schuller, 2018). This factor is related, among other aspects, to social class (Clarke, 2017), family circumstances and gender (Lebert and Voorpostel, 2016). At an organisational level, experiencing a climate of uncertainty in the company induces greater job insecurity among workers, individually (Sora et al., 2013). Therefore, the way to cope with or avoid psychological consequences must be holistic, reaching different levels (Bliese and Jex, 2002; Sora et al., 2011).

Finally, having addressed the relevance of the social support-related strategies to mitigate the effects of job insecurity on health, especially on women’s health, we shall now tackle some intervention measures by way of conclusion. Two associated factors, which would hamper the implementation of this strategy, have been identified. The first is the increase in temporary contracts and more unstable working conditions (Crespo et al., 2009; Burchell et al., 2014; Nielsen et al., 2015). This fact makes it difficult to create those support networks at the workplace. On the other hand, in the economic and cultural context of liberal capitalism that fosters individualism, the individual often views him or herself as responsible or guilty of his or her labour situation (Marzano, 2011; Agulló-Tomás et al., 2018). This increases the use of strategies focused on individual action, or self-criticism as an emotional response. However, our results, in the line of previous studies by Richter et al. (2013) or Gattino et al. (2015), show that these strategies are not very efficient to cope with job insecurity, and that they even deteriorate health further.

### Limitations and Future Research

Some limitations must be considered when interpreting the results of this study. It is a research study with a cross-sectional design, but it would be advisable to analyze these relationships between coping strategies, job insecurity and mental health through longitudinal design studies that allow taking time factors into account, making it easier to obtain causal conclusions. This kind of designs are less common in studies on job insecurity and they are recommended to go deeper into the analysis of this variable (Richter et al., 2013; Cheng et al., 2014), as well as the coping strategies and their effects (Mantler et al., 2005). Moreover, this research study has been performed on a general sample of workers, and in future studies, it would be advisable to control variables such as age, working conditions, type of professional sector, salary, etc., as well as to analyse different worker profiles, or compare different cultural and socioeconomic frameworks. It should also be noted that there is a large presence of partial contracts in the sample, especially in women. It would be necessary to address this aspect in more depth in the future, due to its implications of mental health. This must be done whilst maintaining the gender approach, as some differences have been identified in terms of the influence of the socialisation of men and women on the development and efficacy of the coping strategies. On the other hand, it would be interesting to use tools to measure coping strategies that focus especially on the specific phenomenon of job insecurity. It is proposed to develop scales that include specific actions or strategies to tackle apotential job loss.

Finally, although no evidence has been found to the extent that belonging to a trade union can mediate between job insecurity and psychological well-being (Hellgren et al., 2000), we propose stimulating this kind of studies in the current framework. In recent years, social movements have been forming groups of people with common labour problems that have turned a stressful personal and family circumstance into collective struggles. These are action-focused resources, but which may represent a relevant source of social support in its different forms (informational, affective, instrumental), and thus, it would be very interesting to replicate this research in male and female workers linked to this kind of groups. In this regard, Lee et al. (2018) highlight the need for research that analyses how community intervention can reduce job insecurity or its effects on people.

### Practical Implications

The present study has relevant practical implications when carrying out interventions on the effects of job insecurity that would be useful for work in the field of job counselling, human resource departments, enterprises in general, as well as other social players engaged in community intervention. The main conclusion reached refers to the fact that workers cannot be deemed responsible for not implementing some specific actions when faced with a potential job loss. Intervention should come at different levels, from the individual level to the organisational and social levels, as it has been observed that social support plays a paramount role. Thus, it is important to implement preventive measures to help people have a support network, and also to teach them how to use it. In companies, it is recommended to implement actions that promote a good working climate that would prevent other kinds of disorders related to the workplace (Boada-Grau et al., 2009), as well as to facilitate personal contact among workers. Outside the company, community intervention is a key element to foster the growth of contact networks among individuals in the social sphere. In terms of individual practical implications, the fact that having a social support network is no longer sufficient is gaining relevance; people must also use them. This is the reason why clinical interventions when a person feels unwell or discomfort due to job insecurity should be geared toward the patient learning how to look to his or her environment to obtain emotional, informational, instrumental and affective resources that decrease the perceived stress. Further, we have found that disengagement strategies (social withdrawal, self-criticism, wishful thinking or problem avoidance) are the ones more strongly linked to mental health, and thus, interventions should be aimed, also, at preventing the use of this kind of coping.

## Conclusion

The results shown in this article have broadened the knowledge on the way workers can cope with perceived job insecurity to reduce its negative effects on mental health. It has been found that there are coping strategies that may play a mediating role, with some gender differences. Mainly, disengagement strategies aggravate the psychological consequences of job insecurity, such as, wishful thinking, self-criticism, and problem avoidance in women, and social withdrawal in men. The only strategy that plays a buffering role between insecurity and mental health is social support, and it is only effective in women. We conclude, therefore, by stating that using personal social networks to cope with job insecurity is the best strategy to avoid developing psychological disorders resulting from this situation, especially depressive disorders. We thus propose the need to work on preventing and treating job insecurity from a multilevel or holistic approach, including the individual sphere, but aimed at reinforcing social bonds. This study confirms the already widespread idea that gender roles limit our psychological resources, in this case referring to the way in which we cope with a specific source of stress such as job insecurity. Hence, we underscore the need to promote and develop new and more egalitarian forms of education and socialisation.

## Ethics Statement

This study was carried out in accordance with the requirements and protocols of the Ethics Committee of Oviedo University. The protocol was approved by the Ethics Committee of Oviedo University. All subjects gave written informed consent in accordance with the Declaration of Helsinki.

## Author Contributions

SM-E, JL, and EA-T conceived and designed the work. SM-E, HL, and RS-V collected the data. SM-E, JL, EA-T, JR-S, and RS-V analysed and interpreted the data. SM-E and JL drafted the article. EA-T and JR-S critically revised the article and approved the published version.

## Conflict of Interest Statement

The authors declare that the research was conducted in the absence of any commercial or financial relationships that could be construed as a potential conflict of interest.
